# Organizing pneumonia: a rare complication of a complicated pulmonary hydatid cyst

**DOI:** 10.1590/0037-8682-0335-2023

**Published:** 2023-09-22

**Authors:** Yener Aydin, Hayri Ogul, Ali Bilal Ulas

**Affiliations:** 1 Ataturk University, Medical Faculty, Department of Thoracic Surgery, Erzurum, Turkey.; 2 Duzce University, Medical Faculty, Department of Radiology, Duzce, Turkey.

**FIGURE 1:**
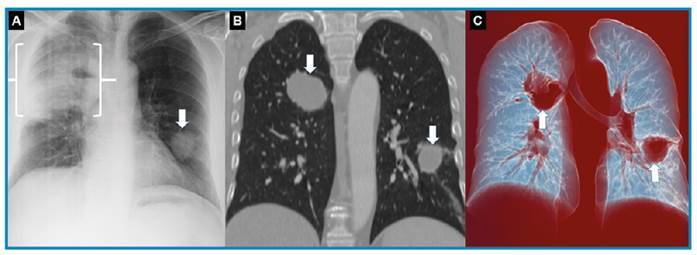
Direct radiography **(A)** showing an area of consolidated pneumonia (bracket) in the right upper zone and a smooth-edged lesion in the left lower zone (arrow). In the parenchymal, the coronal reformatted CT scan **(B)** and air-volume rendering 3D reformation sections **(C)** showing round-shaped lesions in the lower lobe of the left lung and upper lobe of the right lung (arrows).

A 48-year-old man arrived at our facility, presenting a 2-month history of chest pain, cough, low-grade fever, loss of appetite, and fatigue. He had no family history of tuberculosis nor any history of immunosuppressive treatment, alcohol consumption, smoking, drug addiction, or trauma. The Echinococcus IgG ELISA test returned a positive result, while all other laboratory tests were normal. Given the endemic nature of the region and the radiological findings (Figure 1), a preliminary diagnosis of bilateral hydatid cyst was made. The patient underwent a right utility thoracotomy, and an open lung biopsy was performed on the lesion in the upper lobe of the right lung. Histopathological examination confirmed the diagnosis of organizing pneumonia, with no macroscopic evidence of a hydatid cyst membrane. However, a detailed postoperative histopathological examination revealed a hydatid cyst. Consequently, the patient underwent a re-thoracotomy, and the area of organizing pneumonia was excised using a wedge resection.

Hydatid cyst, a parasitic disease, can affect any part of the body^1^. When localized in the lungs, hydatid cysts can cause serious complications upon rupture and may pose diagnostic challenges^2^. Organizing pneumonia, an interstitial lung disease, is characterized by an inflammatory reaction in the alveolar connective tissue, resulting from lung injury. Its etiology encompasses infection, drug toxicity, connective tissue disorders, inhalation injuries, organ transplantation, and radiotherapy^3^. It is crucial to consider pulmonary complicated hydatid cyst as a rare cause of organizing pneumonia.
